# Telemedicine Facilitates CHF Home Health Care for Those with Systolic Dysfunction

**DOI:** 10.1155/2008/235031

**Published:** 2007-10-28

**Authors:** Pennie S. Seibert, Tiffany A. Whitmore, Carin Patterson, Patrick D. Parker, Caitlin Otto, Jean Basom, Nichole Whitener, Christian G. Zimmerman

**Affiliations:** ^1^Idaho Neurological Institute, Saint Alphonsus Regional Medical Center, Boise, ID 83706, USA; ^2^Boise State University, Boise, ID 83725, USA; ^3^Telemedicine Coordinator, Saint Alphonsus Regional Medical Center, Boise, ID 83706, USA; ^4^Corporate Development, Saint Alphonsus Regional Medical Center, Boise, ID 83706, USA; ^5^Neurosurgeon, Saint Alphonsus Regional Medical Center, Boise, ID 83706, USA

## Abstract

An estimated 5 million Americans have congestive heart failure (CHF) and one in five over the age of 40 will develop CHF. There are numerous examples of CHF patients living beyond the years normally expected for people with the disease, usually attributed to taking an active role in disease management. A relatively new alternative for CHF outpatient care is telemedicine and e-health. We investigated the effects of a 6-week in-home telemedicine education and monitoring program for those with systolic dysfunction on the utilization of health care resources. We also measured the effects of the unit 4.5 months after its removal (a total of 6 months post introduction of the unit into the home). Concurrently, we assessed participants' perceptions of the value of having a telemedicine unit. Participants in the telemedicine group reported weighing more times a week with less variability than did the control group. Telemedicine led to a reduction in physician and emergency department visits and those in the experimental group reported the unit facilitating self-care, though this was not significantly different from the control group (possibly due to small sample size). These findings suggest a possibility for improvement in control of CHF when telemedicine is implemented. Our review of the literature also supports the role of telemedicine in facilitating home health care and self-management for CHF patients. There are many challenges still to be addressed before this potential can be reached and further research is needed to identify opportunities in telemedicine.

## 1. INTRODUCTION

The American Heart Association reports that one in five Americans over the age of 40 will develop congestive heart failure (CHF) [1]. This common clinical syndrome is among the top diagnostic-related groups (DRGs), and is a leading cause of hospitalizations and rehospitalization in industrialized countries [2]. Outcomes related to heart failure remain relatively poor, despite advances in pharmacological therapy and medical care [3]. Caring for CHF patients is particularly expensive because of high rates of readmissions. As the vast baby boomer population ages, all age-related illnesses will increase in prevalence [4]. CHF is no exception: its prevalence and high medical resource consumption will continue to increase [5].

CHF is a chronic condition where appropriate disease management is critical [6]. Effective disease management requires the patient to take an active role in his/her health. Indeed, CHF is an area where patient empowerment medicine is of particular importance. Unfortunately, many with CHF do not successfully manage their disease; thus, rehospitalization and high mortality rates prevail. Frequent communication between the patient and health care professionals, intensive education programs, and home health monitoring can help reduce hospitalizations and mortality rates [7]. Despite the dismal prognosis, there are numerous examples of CHF patients living beyond the years normally expected for people with the disease. These successes are usually attributed to patients taking an active role in disease management, facilitated by appropriate diet, exercise, daily self-measurement (e.g., weight scales and blood pressure devices), medication compliance, education, recognition of disease-related symptoms, and support from health care professionals [8–11].

Experts agree that outpatient care is important in achieving the best possible outcomes for patients with CHF, viable forms are currently being investigated and debated [12]. Research has demonstrated that home health nurse visits following discharge can improve CHF outcomes and reduce rehospitalizations. However, individualized care costs are high [13]. Also, the numbers of qualified nurses are diminishing while the need for nurses is growing exponentially. A relatively new alternative for CHF outpatient care is telemedicine and e-health. Studies show that this approach can achieve similar results to that of home health nurse visits [14] by improving outcomes [2, 15] and quality of life [16], and potentially reducing readmission and morbidity rates [17]. Telemedicine offers the added benefit of reducing costs, without sacrificing the level of care [2, 15]. Accordingly, we designed a home-monitoring program to investigate the value added by having a 6-week in-home telemedicine unit for people diagnosed with CHF systolic dysfunction. We also measured the effects of the unit 4.5 months after its removal (a total of 6 months post introduction of the unit into the home). We intended to find a significant difference in the number of calls and visits to the physician, emergency department visits, hospitalizations, and New York Heart Association (NYHA) classification scores postintervention. We also intended to find the home monitoring system increased the number of times the patient weighed themselves per week, compared to the control group.

Further, the potential for telemedicine and e-health is particularly promising for those living in rural areas where health care access is diminished [18]. Most communities in the state of Idaho are considered rural and could particularly benefit from telemedicine and e-health programs. As is the case across the USA, Idaho has a significant number of people with CHF who need assistance in managing their disease.

## 2. MATERIALS AND METHODS

This study investigated the efficacy of a 6-week in-home telemedicine monitoring program, as well as measured whether the program would retain effectiveness during a 6-month period by comparing health care utilization scores. The utilization of health care was determined by the number of times participants contacted and visited physicians, emergency department visits, and hospitalizations. The patients were also assessed based on the number of times they weighed themselves per week, and NYHA scores pre and post experimental period. In addition, participants' perceptions of the value of having a telemedicine unit were also obtained.

Patients who met the criteria (refer to Table 1) and agreed to participate were interviewed by a research nurse at which time the project, confidentiality measures, and informed consent were presented. Each patient was provided a copy of the *Learning to Live with Heart Failure* Self-Care Handbook, an educational booklet describing the disease, possible medications, dietary restrictions, exercise suggestions, therapies, and local support groups. Participants were then assigned to either the telemedicine or control group and given study identification numbers. The control group's active participation ended until the 6-month follow-up survey to calculate the health care utilization score. The experimental group participated in a 6-week monitoring period and answered a follow-up survey 4.5 months after the monitoring period was completed (i.e., 6 months from the start of their participation).

Participants in the telemedicine group were instructed to complete a monitoring session each morning for the 6-week period. A daily monitoring session included weighing on a scale and answering a health questionnaire. If there was a weight gain or loss of ≥ 3 pounds overnight, or a weight gain or loss of ≥ 5 pounds over one week, the participant was prompted to contact their physician. Likewise, alerts were incorporated into the health questionnaire to notify the participant of signs and symptoms that should be reported. No participant weights or responses to the health questions were sent from the monitoring system to the research team. The research team only tracked whether or not the participant interacted with the monitor on a daily basis. The participants were also instructed to call the research nurse if he/she would be unable to complete monitoring sessions for more than 2 days. Queries obtained from the telemedicine unit highlighted participants who had not completed monitoring for 3 or more days. These patients were contacted to determine why monitoring was not completed. Following the 6-week program the equipment was removed from the home and the participant completed a telemedicine satisfaction survey with the research staff.

A chart review was conducted and a phone interview was completed by the research team 6 months from initial start date. Information was obtained regarding the number of times the patient contacted the physician's office, was seen by the physician, was seen in the emergency department, and/or admitted to the hospital. Further information was obtained regarding the number of times a patient weighed themselves a week.

The telemedicine unit was ViTel Net's DataGate system, which included a notebook PC, a touch-screen monitor, and a scale. The notebook PC dimensions were approximately 9.3 inches by 6.9 inches by 2 inches. The monitor dimensions were approximately 12 inches by 9 inches by 3 inches. The A&D LifeSource MD Digital Scale UC-321PL scale was used to measure the weight in 1/10 pound increments, up to a maximum of 450 pounds. The weight value was sent via the RS232 port.

The PC unit connected to a standard electrical wall outlet. The system was very user friendly, including a 12 inch color touch screen with simple point-and-click buttons for presenting educational material, audio prompts, and custom workflow sequences (ViTel Net Description). Once the telemedicine unit was installed in the participant's home, they received the telemedicine participant information packet, which contained instructions for use and care of the equipment, a troubleshooting guide, and research contact information (names and phone numbers) for the research nurse, principal investigator, and project manager. The participant received instruction on the equipment and then was asked to demonstrate appropriate use of the equipment to complete the training session.

## 3. RESULTS

Of the 23 participants, 13 people received the telemedicine program (age 50–81, mean 66.08; 8 male, 5 female; 69% married; 100% Caucasian) and 10 people (age 55–90, mean 71.00; 8 male, 2 female; 80% married; 100% Caucasian) were in the control group. As was our intention, the two groups were similar in age, sex, marital status, years of education, and NYHA classification scores (refer to Table 2).

### 3.1. Comparisons: telemedicine group versus control group

**Figure 1:**
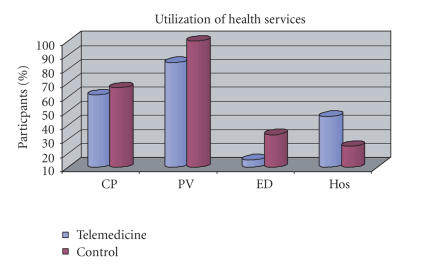
Percent of patients who contacted their physician (CP), visited their physician (PV), utilized the emergency department (ED), and/or were hospitalized (Hos).

We compared telemedicine and control group health care utilization scores using t-tests and chi-square according to variable type and found no statistical significance. Descriptive statistic analysis revealed a pattern of data that suggests the possibility of reaching statistical significance if the sample size were greater. For example, examination of percentages of contacts to physician, physician visits, and emergency department visits conveyed a pattern of higher scores (though not statistically reliable) for the control group (refer to Figure 1 and Table 3). Additionally, people in the telemedicine group reported weighing more times a week with less variability (standard deviation) than did the control group (refer to Table 3). An unexpected result was the higher rate of hospitalizations in the telemedicine group (refer to Table 3). A possible explanation could increase awareness and observation of CHF symptoms, though this was not directly assessed.

### 3.2. Reports from the telemedicine group

We analyzed information regarding the experience of being in the telemedicine group and found that despite nearly half (46%) of the participants reporting no previous experience with computing systems, all but one of the participant reports were positive. The person who did not have a positive experience indicated that “the telemedicine unit made her worry too much about her condition so it stressed her heart.” When asked the open-ended question of what participants liked best about having the telemedicine unit in their home, the majority indicated that the unit helped them be aware of factors important for managing their disease and helped them to control and be more aware of their weight. Seventy-five percent of participants indicated they would like to continue using the unit. Though we are unsure why 25% did not wish to continue, some reported complaints were “repetitive lessons,” “redundant questions,” and “would be more effective if interaction was involved.” The data analysis revealed numerous ways by which the telemedicine unit facilitated self-care. Spearman's rho correlation revealed significant correlates between improved self-care and usage of the telemedicine unit. For example, those who believed that having the telemedicine unit in their home will help them take better care of themselves in the future were highly correlated with better understanding their conditions, *r*_s_ = .854, *P* < .001; and with identifying important symptoms, *r*_s_ = .753, *P* < .001. Those who believed that the unit helped them better understand their condition were highly correlated with taking their medications daily, *r*_s_ = .952, *P* < .001; and limiting their intake of salt, *r*_s_ = .762, *P* < .001;. Also, those who would recommend use of the telemedicine unit were highly correlated with staying healthier, *r*_s_ = .855, *P* < .001; taking medications daily, *r*_s_ = .999, *P* < .001; and calling to seek help, *r*_s_ = .852, *P* < .001. For all correlations concerning facilitation of self-care and perspectives regarding the telemedicine unit, see Tables 4 and 5, respectively.

## 4. DISCUSSION

**Figure 2:**
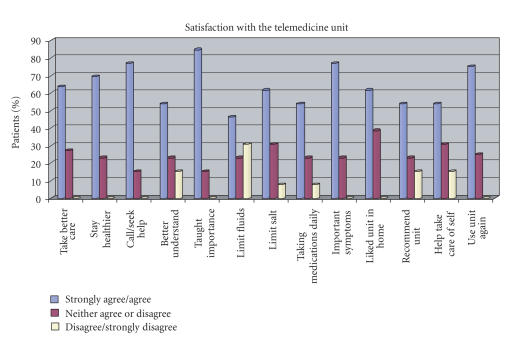
Percent of patients' overall satisfaction with the telemedicine unit and satisfaction in the unit's assistance in managing CHF.

Despite finding no statistically significant results when comparing the experimental and control groups for health care utilization rates, we are encouraged by the reports from those in the telemedicine group indicating the unit facilitated self-care (refer to Figure 2 and Table 5). These reports and our review of the literature support the role of telemedicine in facilitating home health care and self-management for CHF patients. We present these findings in hopes of strengthening future research efforts in telemedicine.

The inclusion/exclusion criteria used in this study resulted in a sample that was probably too small to glean statistically reliable results. For example, we had 105 referrals for participation. Of those, 23 enrolled, 61 were excluded because of not meeting inclusion criteria, and 21 were deceased either before enrollment or before completing the study. Numerous others who would have met the inclusion/exclusion criteria were too ill to participate. Of the 23 enrolled in the study, two were deceased before the study period ended and two more died within a month of ending the study. Of the 61 who did not meet inclusion criteria, most were due to an EF of greater than 40%, CHF was not due to systolic dysfunction, or did not have an echo within six months.

This study was conducted in Idaho, USA, which is considered a rural state and where we hope to better promote the use of telemedicine in rural communities. Though telemedicine seeks to benefit this demographic, distance, and physical disabilities complicate travel, and to hinder research efforts. Due to software limitations, only English speaking patients were included. Additionally, our study consisted of only white participants. Although Idaho is predominately English speaking and white, an accurate model would include other ethnicities.

## 5. FUTURE RECOMMENDATIONS

The largest challenge in many telemedicine studies is obtaining study participants. The difficulties that are achieving adequate enrollment for statistically reliable comparisons indicate that future research designs may benefit from less stringent inclusion and exclusion criteria. Obtaining more patients in less advanced stages of a disease may be one way to improve enrollment. People in all stages of a chronic condition may benefit from home monitoring and increased education. Comparisons of telemedicine's effects at various stages in the disease process may improve data on the efficacy of telemedicine. For example, people in the early stages of a disease may particularly benefit from an early telemedicine intervention, as proper self-care may slow the disease process.

A central goal of telemedicine is to improve delivery of health services to underserved areas. However, enrollment difficulties are exacerbated in studies focusing on rural residents. Chronic conditions inhibit travel and mobility for all patients, regardless of residency location. Therefore, expansion of study demographics to include both urban and rural participants may be another way to facilitate improved enrollment. Future research can also be strengthened with the addition of a multilingual version of the software. This will prevent any biases from restricting the study population. In a future home monitoring project, regarding diabetes management, we will use upgraded equipment from the current study and plan to work with clinics in both rural and urban areas to obtain a larger sample size. This study will provide an opportunity to examine differences and similarities between rural and urban populations in the health benefits achieved through the use of telemedicine.

Having decreased hospitalization as a primary metric and goal may not accurately convey efficacy of telemedicine. It is possible that greater awareness prevented delays in necessary hospital admission; thus, better responsiveness occurred. If so, this would be a positive factor rather than an indication that the telemedicine program was unsuccessful. We recommend this issue to be considered in future research designs.

While home monitoring and education for chronic conditions facilitates improved self-care, people still need increased access to specialty services. In many cases, people in rural areas or people with decreased mobility may not receive care that could improve health. Telemedicine applications that allow specialists to view and communicate with patients remotely may help improve outcomes. Hybrid models of health care delivery, including home monitoring and education, telemedicine access to specialists, and in-person visits may improve patient care and outcomes and help alleviate the problems associated with physician shortages.

## Figures and Tables

**Table 1:** Selection criteria.

*Inclusion criteria*	*Exclusion criteria*
Documented CHF due to systolic dysfunction	CHF was caused by diastolic dysfunction
	Is on renal dialysis
CHF with a New York heart classification (NYHC) of II–IV	Has dementia or another uncontrolled psychiatric disorder that can interfere with his/her ability to participate
Documented EF (ejection fraction) < 40% by echo, nuclear medicine, or CCL within 6 months of enrollment	Anticipated survival from a non-CHF cause is less than 6 months
Can read and speak English	Participated in another heart failure research protocol within the previous 6 months
Has an active phone line in his/her home	Currently receives home health nursing services
	Has had a heart transplant
Is able to give his/her own informed consent	If pregnant or of child-bearing age and is trying to become pregnant
Lives at home; is 18 years or older	Is blind, unable to use his/her upper extremities, or has any physical condition that may inhibit him/her from viewing and/or using a computer screen

**Table 2:** Participant information (means).

	Telemedicine	Control
Age	66 ± 9.1	71 ± 13
Years of education	13.6 ± 3	13.8 ± 1.7
Martial status		
Single	2	0
Married	9	8
Divorced	1	0
Widowed	1	2
New York heart classification, start of study	3.5 ± 0.7	3.11 ± 0.8
New York heart classification, end of study	3.6 ± 0.7	3 ± 0.7

**Table 3:** Control and telemedicine patient utilization of healthcare services.

	*Control group*		*Telemedicine group*	
Contact physician	Number of visits	Percentage	Number of visits	Percentage

	0	37.5 Percentage	0	38.5
	1	25.0 Percentage	1	38.5
	2	12.5 Percentage	4	7.7
	3	12.5 Percentage	5	7.7
	13	12.5 Percentage	9	7.7

Visit physician	Number of visits	Percentage	Number of visits	Percentage

	1	12.5 Percentage	0	16.7 Percentage
	2	37.5 Percentage	1	25.0 Percentage
	3	12.5 Percentage	2	33.3 Percentage
	4	12.5 Percentage	3	8.3 Percentage
	6	12.5 Percentage	5	8.3 Percentage
	9	12.5 Percentage	7	8.3 Percentage

Emergency department	Number of visits	Percentage	Number of visits	Percentage

	1	28.6 Percentage	1	15.4 Percentage

Hospitalized	Number of times	Percentage	Number of times	Percentage

	2	14.3 Percentage	1	23.1 Percentage
	—	—	2	23.1 Percentage

Weigh per week	Mean = 3.81		Mean = 4.94	
	Standard deviation = 3.69		Standard deviation = 2.31	

**Table 4:** Facilitation of self-care correlates.

	*Variables*	*Spearman's rho*
Helped take better care of self	Liked unit in home	0.752^(a)^
	Will help take care of self in the future	0.887^(a)^
	Better understand condition	0.665^(a)^
	Recommend telemedicine unit	0.795^(a)^
	Taking medications daily	0.827^(a)^
	Important symptoms	0.760^(a)^
	Use unit again	0.751^(b)^

Will help take care of self in the future	Liked unit in home	0.911^(a)^
	Better understand condition	0.854^(a)^
	Recommend telemedicine unit	0.924^(a)^
	Limit salt	0.712^(a)^
	Taking medications daily	0.934^(a)^
	Important symptoms	0.753^(a)^
	Call to seek help	0.739^(a)^
	Stay healthier	0.753^(a)^
	Use unit again	0.777^(a)^

Better understand condition	Liked unit in home	0.722^(a)^
	Recommend telemedicine unit	0.928^(a)^
	Limit salt	0.762^(a)^
	Taking medications daily	0.925^(a)^
	Important symptoms	0.622^(a)^
	Call to seek help	0.868^(a)^
	Stay healthier	0.870^(a)^
	Use unit again	0.707^(b)^

^(a)^ *p* < .001;

^(b)^ *p* < .05

**Table 5:** Correlates: perspectives regarding the telemedicine unit.

	*Variables*	*Spearman's rho*
Liked unit in home	Helped take better care of self	0.752^(a)^
	Satisfied with training	0.738 ^(a)^
	Will help take care of self in the future	0.911 ^(a)^
	Better understand condition	0.722 ^(a)^
	Recommend unit	0.890 ^(a)^
	Limit salt	0.648 ^(b)^
	Taking medications daily	0.891 ^(a)^
	Important symptoms	0.775 ^(a)^
	Call/seek help	0.771 ^(a)^
	Stay healthier	0.724 ^(a)^
	Use unit again	0.818 ^(a)^

Recommend unit	Helped take better care of self	0.795 ^(a)^
	Will help take care of self in the future	0.924 ^(a)^
	Better understand condition	0.928 ^(a)^
	Limit salt	0.741 ^(a)^
	Taking medications daily	0.999 ^(a)^
	Important symptoms	0.714 ^(a)^
	Call/seek help	0.852 ^(a)^
	Stay healthier	0.855 ^(a)^
	Use unit again	0.811 ^(a)^

^(a)^ *p* < .001;

^(b)^ *p* < .05

## References

[1] Larson MG, Leip EP, Beiser A (2006). Lifetime risk for heart failure: one in five. http://www.americanheart.org/presenter.jhtml.

[2] Schneider NM (2004). Managing congestive heart failure using home telehealth. *Home Healthcare Nurse*.

[3] Chavey WE, Blaum CS, Bleske BE, van Harrison R, Kesterson S, Nicklas JM (2001). Guideline for the management of heart failure caused by systolic dysfunction—part II: treatment. *American Family Physician*.

[4] Stroetmann KA, Stroetmann VN, Westerteicher C (2003). Implementation of TeleCare services: benefit assessment and organisational models. *Studies in Health Technology and Informatics*.

[5] Driscoll A, Worrall-Carter L, Stewart S (2006). Rationale and design of the national benchmarking and evidence-based national clinical guidelines for chronic heart failure management programs study. *Journal of Cardiovascular Nursing*.

[6] Albert NM, Eastwood CA, Edwards ML (2004). Evidence-based practice for acute decompensated heart failure. *Critical Care Nurse*.

[7] Mueller TM, Vuckovic KM, Knox DA, Williams RE (2002). Telemanagement of heart failure: a diuretic treatment algorithm for advanced practice nurses. *Heart & Lung: The Journal of Acute and Critical Care*.

[8] Inglis SC, Pearson S, Treen S, Gallasch T, Horowitz JD, Stewart S (2006). Extending the horizon in chronic heart failure: effects of multidisciplinary, home-based intervention relative to usual care. *Circulation*.

[9] Havranek EP (2005). Improving the outcomes of heart failure care: putting technology second. *Journal of the American College of Cardiology*.

[10] Goldberg LR, Piette JD, Walsh MN (2003). Randomized trial of a daily electronic home monitoring system in patients with advanced heart failure: the Weight Monitoring in Heart Failure (WHARF) trial. *American Heart Journal*.

[11] Edwards CS (2005). Design and implementation of a comprehensive heart failure management program. *Journal of Healthcare Management*.

[12] Crossen-Sills J, Toomey I, Doherty M (2006). Strategies to reduce unplanned hospitalizations of home healthcare patients: a step-by-step approach. *Home Healthcare Nurse*.

[13] Wheeler EC, Waterhouse JK (2006). Telephone interventions by nursing students: improving outcomes for heart failure patients in the community. *Journal of Community Health Nursing*.

[14] Kashem A, Droogan MT, Santamore WP (2006). Web-based Internet telemedicine management of patients with heart failure. *Telemedicine Journal and e-Health*.

[15] Finkelstein SM, Speedie SM, Potthoff S (2006). Home telehealth improves clinical outcomes at lower cost for home healthcare. *Telemedicine Journal and e-Health*.

[16] Myers S, Grant RW, Lugn NE, Holbert B, Kvedar JC (2006). Impact of home-based monitoring on the care of patients with congestive heart failure. *Home Health Care Management & Practice*.

[17] Louis AA, Turner T, Gretton M, Baksh A, Cleland JGF (2003). A systematic review of telemonitoring for the management of heart failure. *European Journal of Heart Failure*.

[18] Krum H, Tonkin A, Piterman L (2004). Bush telegraph: improving outcomes for rural and remote patients with chronic heart failure. *Australian Family Physician*.

